# Safer Opioid Supply programs: Hydromorphone prescribing in Ontario as a harm reduction intervention to combat the drug poisoning crisis

**DOI:** 10.17269/s41997-024-00979-2

**Published:** 2024-12-10

**Authors:** Rose A. Schmidt, Adrian Guta, Nanky Rai, Andrea Sereda, Emmet O’Reilly, Jessica Hales, Gillian Kolla, Carol Strike

**Affiliations:** 1 Dalla Lana School of Public Health, University of Toronto, Toronto, ON Canada; 2 School of Social Work, University of Windsor, Windsor, ON Canada; 3 Parkdale Queen West Community Health Centre, Toronto, ON Canada; 4 Department of Family and Community Medicine, University of Toronto, Toronto, ON Canada; 5 London InterCommunity Health Centre, London, ON Canada; 6 South Riverdale Community Health Centre, Toronto, ON Canada; 7 Regent Park Community Health Centre, Toronto, ON Canada; 8 Canadian Institute for Substance Use Research, University of Victoria, Victoria, BC Canada

**Keywords:** Opioid, Clinical public health, Substance use, Drug overdose, Program evaluation, Primary healthcare, Opioïdes, Santé publique clinique, Usage de substances, Surdoses de drogues, Évaluation de programmes, Soins de santé primaire

## Abstract

**Setting:**

The crisis of unregulated fentanyl-related overdose deaths presents a significant public health challenge. This article describes the implementation and evaluation of four Safer Opioid Supply programs (SSPs) in Ontario, one in London and three in Toronto.

**Intervention and implementation:**

SSPs aim to curtail overdose fatalities while connecting individuals using drugs to healthcare services. The programs involve a daily dispensed prescription of immediate-release hydromorphone tablets for take-home dosing alongside an observed dose of long-acting opioids like slow-release oral morphine. Implemented within a multidisciplinary primary care framework, these programs emphasize patient-centred approaches and comprehensive health and social support.

**Outcomes:**

In our study conducted in 2020/2021, clients and service providers reported that receiving pharmaceutical opioids through these programs improved the clients’ health and well-being. The regulated supply was reported to lead to decreases in overdose incidents, use of unregulated substances, and criminalized activities. Increased engagement with healthcare and harm reduction services and improvements in social determinants of health, such as food security, were also reported. Despite these positive outcomes, some implementation challenges, including capacity issues and provider burnout, were described by service providers.

**Implications:**

Our findings suggest that the combination of safer supply, wrap-around support, and harm reduction within primary care settings can lead to increased healthcare engagement, HIV/HCV prevention, testing, and treatment uptake, reducing the burden of infectious diseases and overdose risk. SSPs have the potential to meaningfully reduce overdose rates, address the ongoing overdose crisis, and if scaled up, influence population-level outcomes.

## Résumé

**Contexte:**

La crise des décès par surdose liée au fentanyl non réglementé constitue un défi considérable pour la santé publique. Cet article décrit la mise en œuvre et l’évaluation de quatre programmes d’approvisionnement plus sécuritaire (PAS) en opioïdes en Ontario – un à London et trois à Toronto.

**Intervention et réalisation:**

Les PAS visent à réduire le nombre de décès par surdose tout en reliant les personnes qui utilisent des drogues à des services de santé. Ils prévoient quotidiennement une prescription de comprimés d’hydromorphone à libération immédiate à emporter chez soi et une dose d’opioïdes à longue durée d’action, comme la morphine orale à libération lente, prise sous observation. Mis en œuvre dans un cadre de soins primaires multidisciplinaires, ces programmes misent sur des approches centrées sur le/la patient·e et sur un ensemble complet de soutien social et de soins de santé.

**Résultats:**

Dans notre étude, réalisée en 2020–2021, les client·e·s et les prestataires de services ont déclaré que le fait de recevoir des opioïdes pharmaceutiques dans le cadre de ces programmes améliorait la santé et le bien-être des client·e·s. Il a été observé que l’approvisionnement réglementé contribuait à réduire les incidents de surdose, l’utilisation de substances non réglementées et l’activité criminelle. On a également fait état d’un engagement accru dans les services de santé et de réduction des méfaits ainsi que d’améliorations de déterminants sociaux de la santé tels que la sécurité alimentaire. Malgré ces résultats positifs, les prestataires de services ont signalé certaines difficultés de mise en œuvre, notamment des problèmes de capacité et d’épuisement des prestataires.

**Incidences:**

Les résultats de notre étude suggèrent que la combinaison d’un approvisionnement plus sécuritaire, d’un soutien global et de services de réduction des méfaits en milieu de soins primaires peut conduire à un engagement accru dans les soins de santé ainsi que dans la prévention, le dépistage et le traitement du VIH et du VHC, réduisant ainsi le fardeau des maladies infectieuses et le risque de surdose. Les PAS ont le potentiel de réduire de manière significative les taux de surdose, de répondre à la crise actuelle des surdoses et, s’ils sont mis à l’échelle, d’influencer les résultats au niveau populationnel.

## Background and context

The volatile drug supply, consisting primarily of fentanyl and its potent analogues, has led to a surge in overdose fatalities in Canada (Gomes et al., 2021). This crisis of unregulated fentanyl-related overdose deaths is a significant public health challenge. Safer supply programs (SSPs) have emerged as a response to this crisis by providing prescription pharmaceutical opioids dispensed daily for take-home use to those dependent on unregulated opioids who are at high risk for overdose mortality. The establishment of SSPs in Canada was driven, like many effective harm reduction initiatives, by a strong push from harm reduction advocates and practitioners. In 2019, the Canadian Association of People who Use Drugs (CAPUD) released a concept document that proposed multiple models for providing a legal and regulated supply of drugs and emphasized that to reduce overdose mortality, safe supply must be accessible enough to “undercut” the illicit market (Canadian Association of People who Use Drugs, 2019, p. 7). SSPs are a model for the delivery of prescribed safer supply, with programs aiming to reduce the use of unregulated fentanyl, connect individuals to healthcare services, and reduce mortality by preventing overdose (Health Canada, 2019).

Despite their novelty, a substantial body of evidence supports the positive impacts of SSPs, with new evidence continually emerging. Epidemiological and population-level studies demonstrate a reduction in fatal and non-fatal opioid toxicities among active SSP clients (Brothers et al., 2022; Gomes et al., 2022; Lew et al., 2022; Slaunwhite et al., 2024; Young et al., 2022). A study using population-level administrative data in Ontario demonstrated a significant reduction in emergency department (ED) visits, hospitalizations, and associated healthcare costs among Safer Opioid Supply recipients one year after program entry (Gomes et al., 2022). These findings align with program evaluations reporting fewer ED visits and hospitalizations, and improvements in self-reported physical and mental health among current clients (Atkinson, 2023; Haines et al., 2022; Kolla & Fajber, 2023). In qualitative research, clients describe decreased use of unregulated opioids, leading to a decrease in the risk of opioid toxicities, and reduced experience of opioid withdrawal and cravings (Bardwell et al., 2023; Haines & O’Byrne, 2023; Ivsins et al., 2020, 2021; Kolla et al., 2024; McNeil et al., 2022; Schmidt et al., 2023). Providers’ perspectives mirror client-reported outcomes, noting reduced opioid toxicity events and reductions in injection drug use and injection-related complications, as well as overall improvements in client health status (Gagnon et al., 2023; Giang et al., 2023). Participants in SSPs also reported increased personal autonomy, reduced stigma, heightened self-perceived safety, improvements in income security, and decreased involvement in criminal activities (Atkinson, 2023; Bardwell et al., 2023; Haines & O’Byrne, 2023; Ivsins et al., 2020, 2021; Kolla et al., 2024; McNeil et al., 2022; Schmidt et al., 2023). A scoping review of the literature on safer supply found that while program participation was associated with beneficial client outcomes, few large population-level studies had been conducted to date due to the newness of prescribed safer supply, and that these studies would be useful to examine rarer outcomes as well as key issues that have been raised, such as diversion (Ledlie et al., 2024).

Various harm reduction interventions, including overdose prevention sites, naloxone distribution programs, drug-checking technologies, and opioid agonist therapy (OAT), have been implemented and scaled up in Canada to combat the overdose crisis (Strike & Watson, 2019). While evidence demonstrates the effectiveness of these interventions (Irvine et al., 2019), their slow implementation and unequal geographic distribution hinder a comprehensive response to this crisis (Strike & Watson, 2019), which has only been exacerbated by the COVID-19 pandemic (Gomes et al., 2021). There are also significant barriers to access and retention in traditional OAT like methadone, including suboptimal doses in the context of fentanyl, use of urine drug screening in punitive ways, and limited patient control over dosing and administration, including the need to “earn” take-home doses (Bromley et al., 2021). Recognizing the need for an alternative approach to reduce overdose mortality, clinicians in various locations, including Toronto and London, started prescribing safer supply to address their clients’ needs as early as 2016 (Gomes et al., 2022). SSPs offer a harm reduction–based approach to providing an alternative to the volatile street drug supply, recognizing the diverse reasons for opioid use, including managing withdrawal symptoms, experiencing euphoria, and managing pain.

In this article, we describe the implementation and evaluation of four SSPs in Ontario led by members of the author team, one located in London (AS) and three in Toronto (NR, EO, JH). We aim to increase the understanding of SSPs as an important facet of the solution to the complex public health crisis being driven by a volatile supply of unregulated drugs.

### Evaluation design

The description of the programs and primary outcomes is derived from a community-engaged, implementation-focused evaluation of the programs conducted in 2020/2021. The objective of our evaluation was to understand the role of SSP in reducing the risk of overdose and improving the well-being of people dependent on opioids. More details of the study design and data analysis have been published elsewhere (Gagnon et al., 2023; Schmidt et al., 2023). We collected data using qualitative interviews, sociodemographic surveys, and questionnaires about organizational structure and clinical practice. We used a semi-structured interview guide designed to collect data on the implementation process and program outcomes. In total, we collected data from 73 people (*n* = 21 service providers, 52 clients) and four programs. These data represent a snapshot of the programs in 2021 (see Table 1 for a description of program characteristics). CS and AG led the evaluation, and while clinicians were members of the study advisory team, they were not involved in the data collection or analysis.

**Table 1:** Program characteristics, snapshot as of February–March 2021

	InterCommunity Health Centre	Street Health	South Riverdale	Parkdale Queen West (both sites)
City	London	Toronto	Toronto	Toronto
Service delivery location	Community health centre	Non-profit, community-based organization	Community health centre	Community health centre
Number of clients	247	31	46	92
Target number of clients	400	50	250	175
Number of people on waitlist	150	0	13	29
Number of discharges/dropouts (past 6 months)	2	3	25	13
Prescribers				
Physician	1	0	0	4
Nurse practitioner	1	1	1	2
Total	2	1	1	6
Allied health professionals				
Nurse	3	1	1	4^a^
Community health worker	0	1	1	0
Care facilitator/case manager	2	0	0	2
System navigator	1	0	0	2
Outreach/in-reach worker	3	0	0	0
Lab technician	1	0	0	0
Total	10	2	2	8
Wrap-around services				
HIV continuum of care	Yes	No	Yes	Yes
HCV continuum of care	Yes	Yes	Yes	Yes
Housing support	Yes	Yes	Yes	Yes
Income/employment^b^	Yes	Yes	Yes	Yes
Addiction treatment referrals	Yes	Yes	Yes	Yes

^a^Two registered nurses and two licenced practical nurses

^b^Income-related services included support with Ontario Disability Support Program (ODSP) and Ontario Works (OW) applications, liaising with OW/ODSP workers, completing forms for additional income support (e.g. transportation), and supporting clients’ access to resources when faced with eviction (e.g. housing stabilization fund). Employment support included connecting clients with employment services and training/education programs

## Implementation and setting

Ontario’s SSPs were established as a harm-reduction-informed intervention to address limitations in existing harm reduction and addiction treatment amid the worsening toxicity of unregulated drugs.

I think we were all shook by the overdose crisis. And I think once the CTS [Consumption and Treatment Service; a supervised injection site] was opened, we realized that they were never – we always knew they were never going to be enough. (Prescriber)

As one clinician told us, when they started to prescribe hydromorphone off-label for people dependent on opioids, “it was a clinical practice. I was prescribing, just like I would prescribe to any other patient any other medication.” Through collaborative networking among clinicians, prescribing safer supply evolved into a formalized practice, resulting in the publication of a guidance document for implementing new programs (Hales et al., 2020). Pilot funding from Health Canada’s Substance Use and Addiction Program (SUAP) was applied for in 2019 and obtained in 2020 (Health Canada, 2019), which allowed for increasing the number and size of some programs and developing a more comprehensive model to meet the needs of people who use drugs. The College of Physicians and Surgeons of Ontario (CPSO) issued a statement providing guidance for the medical profession in this area in March 2020 (CPSO, 2020).

The four SSPs operate slightly differently, with three integrated into primary care clinics in community health centres (CHC), and the fourth run by a not-for-profit organization providing primary care to people who are unhoused. The goal of these programs is to reduce the risk of overdose and other drug-related harms by offering a safer alternative to the volatile street opioid supply while emphasizing client autonomy and individualized care. Family physicians or nurse practitioners prescribe the medications, with registered nurses, community health workers, and/or system navigators providing wrap-around primary care and referrals/connections to other health and social services. Wrap-around services included full-scope primary care, HIV/HCV testing and treatment, wound care, emergency food, informal counselling and crisis support, support to obtain government identification and apply for income support programs (i.e. disability benefits), harm reduction and overdose prevention education, and referrals to other addiction medicine services. The program in London has a larger multidisciplinary team with access to services both in the SSP and at the CHC where it is delivered, while the Toronto programs, particularly the not-for-profit, are smaller and collaborate with other organizations in the community to provide wrap-around service delivery.

The inclusion criteria for the SSPs are current opioid dependence, current use of opioids from unregulated sources, a history of non-fatal opioid overdose, and previous unsuccessful enrolment in a substance use treatment such as OAT or lack of interest in OAT. The programs also give priority for enrolment to those who are unhoused, have untreated HIV or HCV, are pregnant, and/or self-identify as a woman, a sex worker, Black, Indigenous, or a person of colour, leading to a patient population of those most at risk from drug-related harms.

Intake appointments include a medical and substance use history, a discussion of current drug use patterns, and point-of-care urine drug screening “to confirm that there’s fentanyl present” (Allied Health Provider). During intake, the program structure and client responsibilities as well as the client’s goals for participating in the program are also discussed, as are potential reasons, risks, and program consequences for diverting prescribed medications. These appointments are designed to be manageable, often brief or broken into shorter sessions, considering individuals may be experiencing withdrawal symptoms or sedation from recent drug use. Clients typically receive a prescription during the first appointment, with frequent follow-ups until their dose is titrated and stabilization is achieved.

As we’re titrating up, there’s a lot of patient involvement. ‘How are you feeling in terms of withdrawal, what do you feel that you need to get you through the day, how much are you using at a time?’ People are given a lot more ability to self-judge and self-titrate. We have upper limits on how high we’re willing to go. But otherwise, a lot of the dose adjustments from after that first meeting are a collaborative decision. (Prescriber)

Clients are prescribed immediate-release hydromorphone tablets (brand name Dilaudid) for daily dispensed, take-home use. In Ontario, tablet hydromorphone formulations were used as they dissolve easily for injection and are covered by the provincial formulary. Most clients are also prescribed longer-acting opioids like slow-release oral morphine or methadone, dispensed daily for witnessed ingestion at a pharmacy of the client’s choosing. Clients are provided with education on harm reduction techniques, including preparing tablet hydromorphone for injection for those who choose to inject. Urine drug screening (UDS) is used as part of routine care in alignment with the goals of the program to confirm the presence of hydromorphone to monitor for potential diversion and to allow clients who continue using fentanyl from the unregulated supply to monitor the presence of adulterants (i.e. novel analogues, unregulated benzodiazepines). The identification of unregulated or unprescribed drugs by UDS does not lead to penalties such as discharge or reduction in prescribed opioid dose.

The SSP prescribing model differs significantly from injectable OAT programs where liquid hydromorphone or heroin is prescribed and dispensed in pre-filled syringes for observed consumption in clinic at doses intended to reduce opioid cravings and withdrawal symptoms. SSPs also differ from OAT such as methadone (which is administered orally under observation with limited take-home doses until abstinence from unregulated/unprescribed drugs and clinical stability is achieved) or buprenorphine (which does not induce euphoria). The SSP approach also notably differs from OAT as it allows clients to manage dosing and choose their preferred administration route (e.g. oral, injection). One of the programs includes an observed arm, where ingestion of the doses prescribed to individuals at heightened risk for overdose from concurrent heavy alcohol and/or benzodiazepine use is witnessed multiple times a day by a registered nurse at the CHC’s supervised consumption facility. For higher-risk clients in the other programs, a combination of slower titration of medications, lower doses of take-home medications, or witnessed ingestion at the pharmacy is employed.

## Client outcomes

During study interviews, clients and service providers reported positive outcomes for client health and well-being. More details of the primary outcomes of the evaluation have been published separately (see Gagnon et al., 2023; Schmidt et al., 2023). Access to a regulated opioid supply was reported by both clients and providers to lead to decreases in overdose incidents, use of unregulated substances, and criminal activities. Notably, nearly all clients and healthcare providers reported a reduced risk of overdose mortality among clients due to having access to known doses of pharmaceutical opioids, citing a significant reduction in overdose incidents since enrollment. As one client expressed, “I can’t say enough positive things about this program. It’s saved my life; it’s saved countless friends’ lives.” Many clients reported they had reduced or stopped using unregulated drugs and/or reduced or stopped injecting drugs, instead using all doses of prescribed safer supply orally.

Healthcare providers and clients also reported improved health outcomes and increased engagement with healthcare and harm reduction services, with SSPs facilitating increased trust in and reintegration with the healthcare system for individuals who had previously encountered stigma and discrimination. As a result, many clients were willing to address previously unattended health problems. Increased engagement with healthcare and harm reduction services also led to improved HIV/HCV prevention, testing, and treatment access. The SSPs helped clients address many health and social needs in a single location, improving accessibility and knowledge of services. Clients and providers also noted many improvements in the client’s social determinants of health, such as housing stability and food security, as well as less involvement in street-based income-generation activities such as sex work:

I don’t have to work the streets anymore. For anything. Now I can work on things because I have the time. I’m never starving, I’m never in need. Now, I do a pill to get a pill in me. It’s not a whole rigamarole where I have to think about what I’m going to do to get the money, to get the pill, and then worry about feeling guilty about what I had to do and then try to find a spot to do it. None of that happens anymore. (Client)

Many clients found that take-home doses provided the ability to control their medication dosing and allowed them to manage withdrawal symptoms and chronic pain more effectively than with previous treatments like OAT. These synergistic outcomes—improved control over withdrawal symptoms, reduced use of unregulated opioids, reduced engagement in criminalized activities, increased engagement in healthcare, improved health outcomes, and greater social stability—led to significant improvements in the clients’ lives.

They often tell me how much it’s impacted them, which I am very fortunate to be able to get to hear and see first-hand, just the impact that we are having. Folks that might not have seen a doctor for years, if ever, are finally getting complex and concerning medical issues addressed and mental health support and housing support. (Allied health provider)

Clients and providers rarely described negative outcomes associated with the SSP, despite direct questions probing this. When asked, most people said “no” or they could not think of any. The most commonly reported negative impact was the time required to attend appointments and go to the pharmacy for daily dispensed medication. While no participant or prescriber described a specific instance of a client experiencing increased violence, it was presented by a few as a hypothetical risk (e.g. being “jumped” for their prescription). Insufficient program capacity to meet the levels of community need was also raised as an issue. For some clients, having a friend or family member who was unable to access the program elicited feelings of guilt and concern for their well-being, with some clients highlighting that they may share medications with people unable to access the program and experiencing withdrawal or frequent overdose in an attempt to assist them.

## Implementation barriers and program limitations

Despite promising results, program implementation has not been without its challenges. Service providers described challenges including insufficient program capacity, small staff teams, a limited number of prescribers, time-limited program funding, and provider burnout from addressing the complex, time-consuming needs of clients. Many of these challenges were related to the program’s reliance on pilot funding. Many service providers and clients wanted a wider range of medications not available on the Ontario drug formulary, including injectable hydromorphone, diacetylmorphine (heroin), and fentanyl, as well as medications that could be smoked. The lack of stimulant safer supply options was also identified as a challenge. The medicalized structure of the existing programs (e.g. prescribed in primary care) was also identified as a limitation as many people who use drugs distrust healthcare institutions due to negative past experiences.

Additionally, providers told us that the lack of understanding of the goals of the program and a focus on concerns about diversion from their professional and the broader community was challenging, particularly due to the overall focus on abstinence-based models within the current addiction treatment system.

Because for a lot of providers – and I absolutely understand this – some of these prescriptions are frightening to write if you’ve never done it before. And if you don’t understand how these prescriptions are life-saving, life-changing, they can be hard to wrap your mind around them if you are heavily invested or surrounded by very abstinence-based models of substance use care. (Prescriber)

## Implications

Our results correspond to existing evidence and clinical experience that prescribed safer supply programs can lead to important health benefits for people at high risk of overdose death from their use of unregulated opioids. Studies have demonstrated they can significantly improve health indicators (Ledlie et al., 2024); increase healthcare engagement (Haines & O’Byrne, 2023; Schmidt et al., 2023); improve HIV/HCV prevention, testing, and treatment uptake (Gagnon et al., 2023; Schmidt et al., 2023); reduce overdose risk (Brothers et al., 2022; Gomes et al., 2022; Lew et al., 2022; Young et al., 2022); and significantly reduce all-cause and overdose mortality (Slaunwhite et al., 2024).

Our results triangulate with existing research using administrative health data from clients of the London SSP (Gomes et al., 2022). Following entry into the program, participants experienced significant decreases in ED visits (by − 13.9 visits per 100 individuals, 95% CI: − 25.6 to − 2.1), inpatient hospital admissions (reducing by 5.2 admissions per 100 individuals, 95% CI: − 8.7 to − 1.7), and healthcare costs (decreasing by $922 per person, 95% CI: − $1577 to − $268) which were not identified among the matched group of people with opioid use disorder unexposed to safer supply (Gomes et al., 2022).

While the Ontario model of SSPs is a promising intervention with documented benefits for individuals receiving prescribed safer supply, some challenges impact SSPs’ ability to have a broader public health impact. Issues with funding, limited program capacity, limited political, professional and community support, and strong ideological opposition to these programs have hindered their expansion and continue to threaten sustainability. The time-limited federal funding for these programs poses a significant challenge to their sustainability, as provincial governments have been reluctant to provide funding. Several different models of SSP are being scaled up in Canada. The model we describe—situating SSPs in primary care with wrap-around social support—is labour-intensive. Research in Ontario conducted before the federal funding which allowed for program expansion suggested that prescribed safer supply was reaching a population of people who use drugs experiencing significant medical complexity, including multiple previous attempts at OAT (Gomes et al., 2022; Young et al., 2022). These programs continue to prioritize admission to people who experience or are at risk of experiencing significant harm from drug use and a high level of support is necessary to address their complex medical and social needs.

The Ontario SSP model described here demonstrates how safer supply prescribing could be scaled up within existing primary care settings, providing options for the provision of care for people who use drugs outside of an addiction medicine framework. Given the urgency of scaling up service provision amid a devastating drug toxicity crisis in Canada, and research documenting that people with opioid use disorder with a primary care physician were more likely to receive appropriate preventive care, access to safer supply integrated within primary care settings has the potential to improve health outcomes for an often-complex population (Spithoff et al., 2019). In the context of expanding safer supply prescribing more broadly within primary care, many physicians express apprehension about prescribing high opioid doses, given their historical association with opioid over-prescribing (Tyndall & Dodd, 2020). However, simplifying the current crisis to a single cause is reductive and obfuscates how opioid agonist treatments and prescribed opioids are contributing to 10% of drug toxicity deaths whereas fentanyl from the unregulated market is responsible for close to 90% of drug toxicity deaths in Ontario (Gomes et al., 2023). A review of administrative health data in Ontario found that deaths were very rare (≤ 0.016 per person-year) among patients prescribed high doses of immediate-release hydromorphone as safer supply (Young et al., 2022). Additionally, evidence indicates that stringent restrictions on opioid prescriptions have inadvertently driven up the demand for unregulated opioids (Urbanoski et al., 2022).

Opposition to these programs, particularly from some in the field of addiction medicine and conservative governments, has expanded since our evaluation (Duthie et al., 2022; Tyndall & Dodd, 2020; Urbanoski et al., 2022). Vocal opposition to prescribed safer supply, as well as to broader harm reduction interventions, has been described as a “moral panic” due to the focus on unsubstantiated claims and anecdotal reports, and neglect of published research evidence showing positive impacts (Ledlie et al., 2024; Michaud et al., 2024). Stigma remains a significant obstacle to the sustainability and expansion of harm reduction approaches. Diversion is frequently cited as a concern. While the potential for diversion exists, as it does for the prescription of any potentially harmful medication, our findings highlight the multifaceted health and social stabilizing impact of SSPs which contributes to the overall risk–benefit assessment that prescribers must consider (Duthie et al., 2022). Research on safer supply has found diversion is low and commonly motivated by compassionate care (e.g. reducing the overdose risk of a loved one) or trading/selling medications for unregulated opioids, particularly when prescribed doses do not adequately manage withdrawal (Bardwell et al., 2021). These motivations for diversion are best addressed by expanding programs and providing clients with preferred substance types and formulations (Fischer & Robinson, 2023). Nonetheless, the continuous monitoring of population-level health impacts, both positive and negative (e.g. increase in hydromorphone-related deaths among people both prescribed and not prescribed safer supply), is crucial. However, there is a danger of a form of drug exceptionalism being applied to SSP, where safer supply programs are subject to different standards from those applied to other public health interventions intended to reduce an extreme risk of death (Fischer & Robinson, 2023). The politicization of safer supply hampers effective monitoring and adjustment by shifting attention away from scientific evidence and ongoing surveillance.

Expansions of harm reduction and diverse models of safer supply distribution are necessary to address the risk environment and reduce opioid overdose fatalities. Safer supply programs are part of a larger complement of services in the care continuum required to fully address both the high risk of overdose deaths and ongoing systemic social issues related to poverty and criminalization that impact the health of people who use drugs. Aligning safer supply medication options with individuals’ preferences, such as prescribing substances suitable for preferred methods of consumption like inhalation, is important given the increased prevalence of inhalation among people who use unregulated fentanyl (MacDonald et al., 2023). There is also a necessity for non-medicalized avenues to access opioids with known doses and potency, including compassion clubs—which offer access to substances of known doses and composition outside a medical model, and the decriminalization or legalization of drugs (Kalicum et al., 2024; Nyx & Kalicum, 2024; Seliga, 2022). These measures can further mitigate the risk of opioid overdoses, as not all individuals at risk or succumbing to drug poisonings from the volatile street supply of drugs meet the criteria for opioid use disorder or SSP enrollment (Gomes et al., 2023; Seliga, 2022).

## Conclusion

The crisis of volatile street drugs in Canada requires innovative strategies to address the ongoing physical and social harms associated with unregulated opioids. By providing regulated pharmaceuticals of known potency and composition, SSPs can reduce overdose risk and improve health outcomes for people dependent on opioids from the unregulated market and for whom existing substance use programs have not been effective. If scaled up across Canada as part of a larger strategy, the SSP model may reduce population-level overdose rates and help curb Canada’s devastating overdose and drug poisoning crises.

## Implications for policy and practice

What are the innovations in this policy or program?

- SSPs are a novel and promising addition to overdose prevention strategies in Canada.
- These programs are innovative as they provide off-label prescriptions for legal and regulated opioid medications as an alternative to unregulated opioids, to reduce overdose risk and improve health outcomes.
- The programs adapt traditional harm reduction models, challenging abstinence-based practices and offering a fresh approach to this crisis.
- SSPs prioritize client autonomy and employ a multidisciplinary team for comprehensive care, diverging from conventional treatment approaches.
- SSPs represent a paradigm shift by acknowledging diverse reasons for opioid use and addressing systemic issues, marking an innovative response to the complex public health crisis.

What are the burning research questions for this innovation?

- What are the long-term impacts of SSPs on participant outcomes?
- How can continuous monitoring and surveillance systems be established to track both positive and negative population-level health impacts over time?
- How can diverse models of SSPs be developed and tested to address the unique needs of various populations, ensuring inclusivity and accessibility?
- What policy changes and advocacy efforts are needed to secure sustained funding for SSPs, and how can political and professional support be garnered for their expansion?

## Data Availability

Not applicable.
